# Genome-Wide Identification and Analysis of *MKK* and *MAPK* Gene Families in *Brassica* Species and Response to Stress in *Brassica napus*

**DOI:** 10.3390/ijms22020544

**Published:** 2021-01-07

**Authors:** Zhen Wang, Yuanyuan Wan, Xiaojing Meng, Xiaoli Zhang, Mengnan Yao, Wenjie Miu, Dongming Zhu, Dashuang Yuan, Kun Lu, Jiana Li, Cunmin Qu, Ying Liang

**Affiliations:** 1 College of Agronomy and Biotechnology, Southwest University, Chongqing 400715, China; wangzhencq@swu.edu.cn (Z.W.); wyywinyuan@swu.edu.cn (Y.W.); mxj6509@email.swu.edu.cn (X.M.); zxl19930619@email.swu.edu.cn (X.Z.); yaomn3367@email.swu.edu.cn (M.Y.); milk11@email.swu.edu.cn (W.M.); dm1361613848@email.swu.edu.cn (D.Z.); yds5206@email.swu.edu.cn (D.Y.); drlukun@swu.edu.cn (K.L.); ljn1950@swu.edu.cn (J.L.); 2 Academy of Agricultural Sciences, Southwest University, Chongqing 400715, China; 3 Chongqing Engineering Research Center for Rapeseed, Chongqing 400715, China

**Keywords:** MKKs, MAPKs, growth and development, abiotic stresses, interaction, *Brassica napus*

## Abstract

Mitogen-activated protein kinase (MAPK) cascades are common and conserved signal transduction pathways and play important roles in various biotic and abiotic stress responses and growth and developmental processes in plants. With the advancement of sequencing technology, more systematic genetic information is being explored. The work presented here focuses on two protein families in *Brassica* species: MAPK kinases (MKKs) and their phosphorylation substrates MAPKs. Forty-seven MKKs and ninety-two MAPKs were identified and extensively analyzed from two tetraploid (*B. juncea* and *B. napus*) and three diploid (*B. nigra*, *B. oleracea*, and *B. rapa*) *Brassica* species. Phylogenetic relationships clearly distinguished both MKK and MAPK families into four groups, labeled A–D, which were also supported by gene structure and conserved protein motif analysis. Furthermore, their spatial and temporal expression patterns and response to stresses (cold, drought, heat, and shading) were analyzed, indicating that *BnaMKK* and *BnaMAPK* transcript levels were generally modulated by growth, development, and stress signals. In addition, several protein interaction pairs between BnaMKKs and C group BnaMAPKs were detected by yeast two-hybrid assays, in which BnaMKK3 and BnaMKK9 showed strong interactions with BnaMAPK1/2/7, suggesting that interaction between BnaMKKs and C group BnaMAPKs play key roles in the crosstalk between growth and development processes and abiotic stresses. Taken together, our data provide a deeper foundation for the evolutionary and functional characterization of *MKK* and *MAPK* gene families in *Brassica* species, paving the way for unraveling the biological roles of these important signaling molecules in plants.

## 1. Introduction

Plants have evolved specialized mechanisms that sense adverse environmental conditions and adapt their physiology, growth, and developmental processes accordingly through the transcriptional, translational, and post-translational regulation of their enzymatic activities. Protein phosphorylation is the most common post-translational modification observed across organisms and typically involves protein kinases in signaling cascades [1]. Mitogen-activated protein kinase (MAPK) cascades are common signal transduction pathways in all eukaryotes [2] and play critical roles in signal transduction [3,4,5]. In plants, MAPKs transduce extracellular stimuli to induce a range of cellular responses, specifically post-translationally modifying proteins to modulate cellular functions associated with various physiological, developmental, and phytohormonal responses [4,6,7,8]. MAPK signaling cascades also result in the activation of cytoskeletal proteins, phospholipases, and microtubule-associated proteins, and can target transcription factors to modulate the expression of specific sets of genes in response to environmental stimuli [9,10,11].

A canonical MAPK signaling network is composed of three specific protein kinases, namely MAPK kinase kinases (MAPKKKs), MAPK kinases (MKKs), and MAPKs, which are activated sequentially by phosphorylation at conserved activation sites [1,7]. MAPKKKs are the first component in these phosphorelay cascades and phosphorylate and activate MKKs on S/T-X_3–5_-S/T motifs. All MKKs also have a putative MAPK docking domain (D domain) K/R-K/R-K/R-X_1–6_-L-X-L-/V/I in their *N* termini. MKKs are dual-specificity kinases that activate their downstream MAPKs through phosphorylation of the T-X-Y motif in the activation loop (T-loop) [4]. Activated MAPKs then lead to the phosphorylation of various downstream substrates, including transcription factors and other signaling components that regulate the expression of downstream genes [1]. A total of 10 MKKs and 20 MAPKs have been characterized in *Arabidopsis thaliana*, which revealed that *Arabidopsis* MKKs may activate different MAPKs and hence participate in signal integration from distinct signaling pathways [7,12]. MKKs can be classified into four major groups (A–D) based on the sequence of their S/T-X_3–5_-S/T motif and D domain. Similarly, MAPKs also form four groups (A–D) based on their conserved T-X-Y motif, with groups A–C harboring the T-E-Y sequence and group D the T-D-Y sequence [7].

Extensive studies in plants have revealed that MAPK cascades are indispensable for the regulation of the cell cycle, growth, and development, and responses to biotic and abiotic stresses, such as pathogen infection, salt, drought, heat, low temperature, reactive oxygen species (ROS), and heavy metals [8,13,14,15,16,17]. To date, several MAPK signaling pathways have been characterized in detail. The *Arabidopsis* MAPKKK1-MKK4/5-MAPK3/6 module was the first identified MAPK signaling module in plants, mediating the transcriptional upregulation of genes encoding the transcription factors WRKY22 and WRKY29 in response to attacks by both fungal and bacterial pathogens [18,19]. Additional modules have since been linked to the regulation of multiple and varied biological processes. For instance, the MKK4/5-MAPK3/6 module has been shown to play important roles in responses to biotic stress and during stomatal development [18,20,21]. The MKK1-MAPK3/6 and MKK6-MAPK4 branches are also key players in stomatal patterning and dynamics [13,20,21,22]. The MKK3-MAPK8 pathway mediates Ca^2+^ and ROS signaling during early wounding [23]. The MAPKKK1-MKK1/2-MAPK4 branch positively regulates defense responses against necrotrophic fungi, while negatively regulating defenses against biotrophic pathogens; this branch is also activated by drought, freezing, and salt stresses [24,25,26,27]. The MKK9-MAPK3/6 pathway was reported to regulate ethylene signaling and camalexin biosynthesis and might also play a role during leaf senescence [28,29,30]. The MKK6-MAPK4/11 pathway was shown to directly regulate cytokinesis and mitosis [31,32,33,34]. Another example is the abscisic acid (ABA)-activated MAPKKK17/18-MKK3-MAPK1/2/7/14 pathway, which transduces the ABA signal to regulate the expression of a number of ABA-dependent genes [35]. In previous work, we reported that overexpression of the rapeseed (*Brassica napus*) *BnaMAPK1* gene increased plant resistance to drought stress and to the pathogenic fungus *Sclerotinia sclerotiorum* [36,37]. In contrast to A and B group MAPKs, reports on C groups are scarce. Fortunately, the sequence availability of multiple *Brassica* genomes is now allowing a thorough investigation of MKKs and MAPKs and their possible interactions.

Here, we identified and classified MKKs and MAPKs in five *Brassica* species and investigated the expression patterns of *BnaMKK* and *BnaMAPK* in spatial and temporal expression patterns and in response to abiotic stresses. Furthermore, Y2H assays were performed on the potential interaction relationship between the encoded BnaMKKs and C group BnaMAPKs, revealing an important regulation of BnaMKK-BnaMAPK signaling modules in the complex crosstalk between growth and developmental processes and abiotic stress responses. This work thus suggests a molecular strategy to coordinate growth and development and stress responses in *B. napus* and lay a solid foundation for further deciphering MAPK cascades and for future improvement of various defense responses using these signaling pathways for *B. napus* breeding.

## 2. Results

### 2.1. Identification of 47 MKK and 92 MAPK Genes in Five Brassica Species

A systematic Basic Local Alignment Search Tool for Protein (BLASTP) search was performed using the *Arabidopsis* MKK and MAPK protein sequences as query against the genome databases for *B. juncea* (*Bju*), *B. napus* (*Bna*), *B. nigra* (*Bni*), *B. oleracea* (*Bol*), and *B. rapa* (*Bra*). 47 *MKK* genes were identified: 11 genes in *B. juncea*, 14 in *B. napus*, seven in *B. nigra*, six in *B. oleracea*, and nine in *B. rapa* (Table 1). Similarly, 92 *MAPK* genes were identified, with 25 genes in *B. juncea*, 29 in *B. napus*, 18 in *B. nigra*, six in *B. oleracea*, and 14 in *B. rapa* (Table 1). Not all *Arabidopsis MKK* and *MAPK* genes had clear orthologs in the other species: Orthologs for *AtMKK2/9* or *AtMAPK18/19* in *B. juncea*; *AtMKK7* or *AtMAPK3/5/11/12* in *B. napus*; *AtMMK7/8/9* or *AtMAPK4/8/14/17/18* in *B. nigra*; *AtMKK3/5/7/10* or *AtMAPK1/2/3/5/7-16/20* in *B. oleracea*; and *AtMKK2/7* or *AtMAPK6/7/11/14/16/18/20* in *B. rapa* were not found. These *Brassica MKK* and *MAPK* genes were named according to their closest homologs in *Arabidopsis*, and multiple copies of one gene were named with 1, 2, or 3 (details in Table S1). This analysis indicated that the duplication and retention of *MKK* and *MAPK* genes were more frequent in tetraploids than in diploids.

### 2.2. Phylogenetic Analysis of MKKs and MAPKs from Arabidopsis and Five Brassica Species

**Figure 1:**
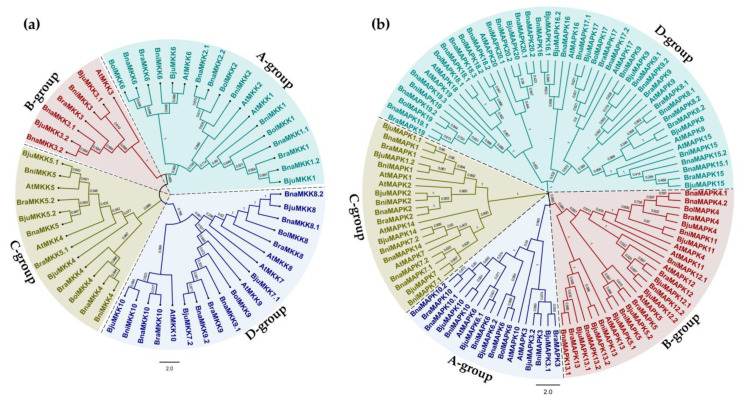
Phylogenetic analysis of MKKs and MAPKs family members from *Arabidopsis thaliana* (*At*), *Brassica juncea* (*Bju*), *Brassica napus* (*Bna*), *Brassica nigra* (*Bni*), *Brassica oleracea* (*Bol*), and *Brassica rapa* (*Bra*). (**a**) Phylogenetic trees of MKKs from *Arabidopsis* and five *Brassica* species. (**b**) Phylogenetic trees of MAPKs from *Arabidopsis* and five *Brassica* species. The unrooted phylogenetic trees were constructed using MEGA-X with the NJ method; the bootstrap test was performed with 1000 iterations. Different colored backgrounds indicate different groups.

To clarify the evolutionary relationships of MKKs and MAPKs in *Arabidopsis* and other *Brassica* species, multiple protein alignments of the full-length MKK and MAPK sequences were performed to generate unrooted phylogenetic trees with the Neighbor-joining (NJ) method. Both multiple alignments and the phylogenetic analysis suggested that MKK proteins were classified into four groups: MKK1/2/6 (A group), MKK3 (B group), MKK4/5 (C group), and MKK7/8/9/10 (D group) (Table 1). Likewise, MAPK proteins were also classified into four groups: MAPK3/6/10 (A group), MAPK4/5/11/12/13 (B group), MAPK1/2/7/14 (C group), and MAPK8/9/15/16/17/18/19/20 (D group) (Table 1, Figure 1). Notably, BraMKK5.1 clustered more closely with AtMKK4 and BjuMKK7.2 with AtMKK9 (Figure 1a). Furthermore, the four MAPK10 proteins BnaMAPK10, BraMAPK10, BjuMAPK10, and BniMAPK10 grouped more closely with AtMAPK6, while BniMAPK11 and BjuMAPK11 were more closely related to AtMAPK14 in the phylogenetic tree (Figure 1b). These results suggest that these proteins in the five *Brassica* species and *Arabidopsis* are closely related, but also imply that functional differentiation may have occurred within multiple groups.

We next focused on *MKK* and *MAPK* genes from *B. napus*. The length of *MKK* coding sequences in *B. napus* covered a wide range, from 735 bp to 1557 bp, with a mean length of 1072.5 bp. The associated molecular weight (MW) across BnaMKK proteins varied from 27.1 kDa to 57.5 kDa, with a mean of 39.5 kDa; the isoelectric point (pI) ranged from 5.4 to 9.4, with a mean of 6.7 (Table S2). Similarly, the length of *BnaMAPK* coding sequences varied from 1110 bp to 1977 bp, with a mean length of 1505.3 bp. The mean MW of BnaMAPKs proteins was 56.9 kDa (range 42.3 kDa to 74.3 kDa), while pI values ranged from 4.8 to 9.3, with a mean of 7.1 (Table S2).

### 2.3. Gene Structure and Conserved Motif Analysis of BnaMKKs and BnaMAPKs in B. napus

**Figure 2:**
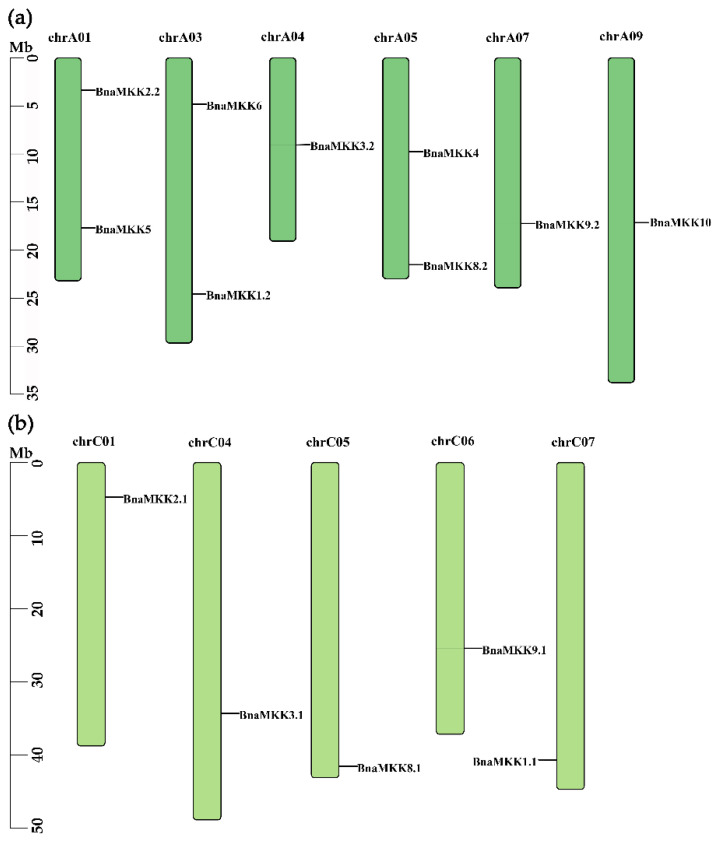
Chromosomal locations of *BnaMKK* genes in *B. napus* genome. (**a**) Genomic position of *BnaMKK* genes in subgenome A. (**b**) Genomic position of *BnaMKK* genes in subgenome C.

**Figure 3:**
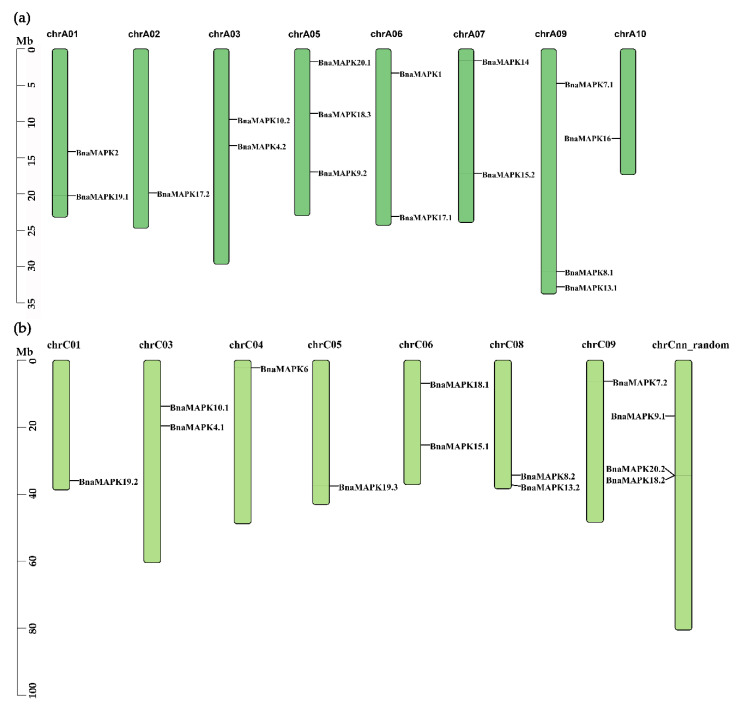
Chromosomal locations of *BnaMAPK* genes in *B. napus* genome. (**a**) Genomic position of *BnaMAPK* genes in subgenome A. (**b**) Genomic position of *BnaMAPK* genes in subgenome C.

The genomic position of all *BnaMKK* and *BnaMAPK* genes were determined to better understand their chromosomal distribution in *B. napus*, an allopolyploid derived from a cross between the diploid species *B. rapa* (A subgenome progenitor) and *B. oleraceae* (C subgenome progenitor). Nine *BnaMKK* genes mapped to the A subgenome (Figure 2a); the remaining five *BnaMKK* genes mapped to the C subgenome (Figure 2b). In the case of the *BnaMAPK* gene family, 16 *BnaMAPKs* mapped to the A subgenome (Figure 3a) and 13 *BnaMAPK* genes mapped to the C subgenome, of which *BnaMAPK9.1*, *BnaMAPK18.2*, and *BnaMAPK20.2* were located to the Cnn_random chromosome, a collection of unmapped scaffolds tentatively assigned to the C subgenome (Figure 3b).

**Figure 4:**
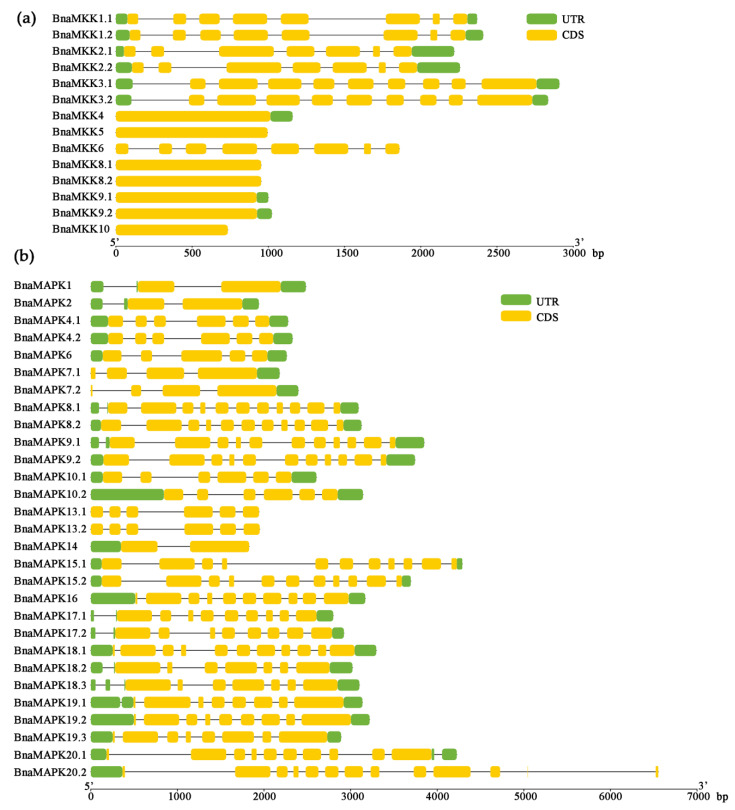
Gene structures of *BnaMKK* and *BnaMAPK* genes. (**a**) Exon–intron organization of *BnaMKK* genes. (**b**) Exon–intron organization of *BnaMAPK* genes. The untranslated regions (UTRs) are shown as green blocks, exons as yellow blocks, and introns as thin black lines.

Next, the structures of *BnaMKK* and *BnaMAPK* genes were examined. *BnaMKK* genes had between one and nine exons, with seven *BnaMKK* genes (*BnaMKK4*, *BnaMKK5*, *BnaMKK8.1*, *BnaMKK8.2*, *BnaMKK9.1*, *BnaMKK9.2*, and *BnaMKK10*) consisting of a single and very long exon, much longer than any other exon in the other *BnaMKK* genes (Figure 4a). The number of exons among *BnaMAPK* genes varied from two to 13, with three C group members (*BnaMAPK1*, *BnaMAPK2*, and *BnaMAPK14*) having only two exons (Figure 4b).

**Figure 5:**
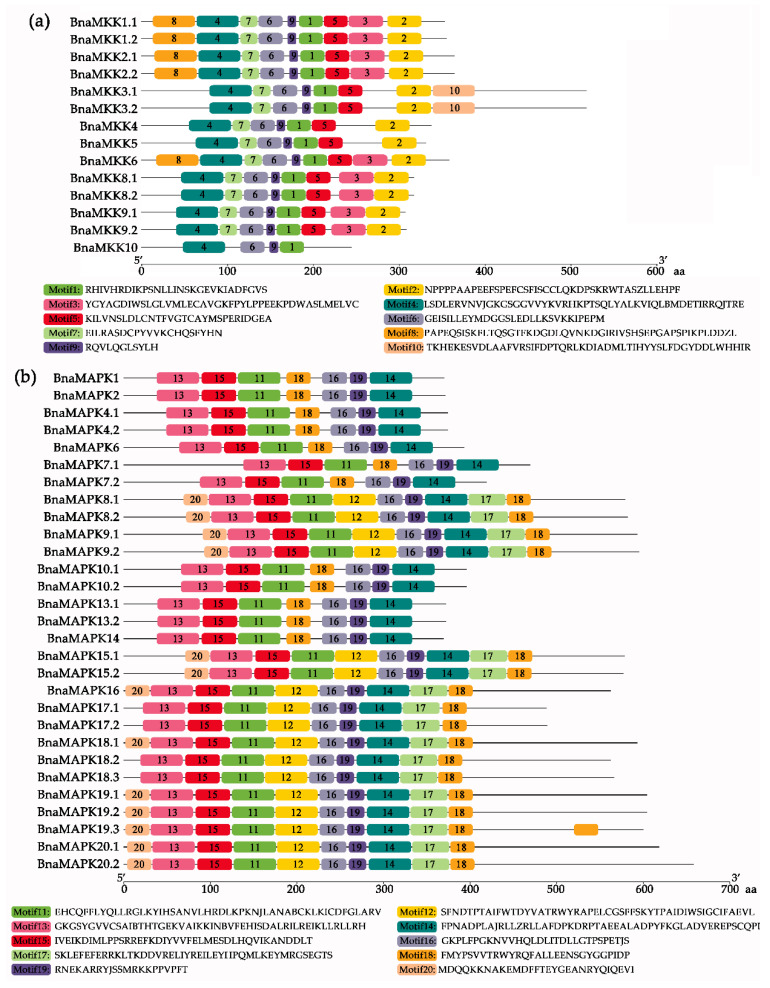
Distribution of conserved motifs in BnaMKK and BnaMAPK proteins. (**a**) Conserved motifs of BnaMKKs. (**b**) Conserved motifs of BnaMAPKs. Each motif is represented by rectangular boxes with different colors.

Then, the conserved motifs of BnaMKK and BnaMAPK proteins were predicted by the Multiple Expectation Maximization for Motif Elucidation (MEME) suite. The number of discoverable motifs was set to ten for both BnaMKKs and BnaMAPKs. Motif 4 in BnaMKK proteins is only present in the PLN00034 superfamily of proteins and defines bona fide MKKs; another motif presents in both BnaMKKs and BnaMAPKs was related to the catalytic domain of serine/threonine-specific and tyrosine-specific protein kinases (PKc-like) (Figure 5). All BnaMKK family members shared motifs 1, 4, 6, and 9, while motifs 2, 5, and 7 were shared among all BnaMKK members with the sole exception of BnaMKK10. Motif 10 was only identified in BnaMKK3.1 and BnaMKK3 (Figure 5a). From a similar analysis on BnaMAPK family members, motifs 11, 13, 14, 15, 16, 18, and 19 were detected in all BnaMAPK members, with motif 18 in D group BnaMKKs located C-terminal to motif 17, and motifs 11, 15, 16, and 19 located between motif 13 and motif 14 in each protein (Figure 5b). The clear divergence in *BnaMKK* and *BnaMAPK* gene structures and motif composition of their encoded proteins may indicate functional differentiation.

### 2.4. Expression Patterns of BnaMKK and BnaMAPK Genes in Different Tissues and in Response to Abiotic Stress

**Figure 6:**
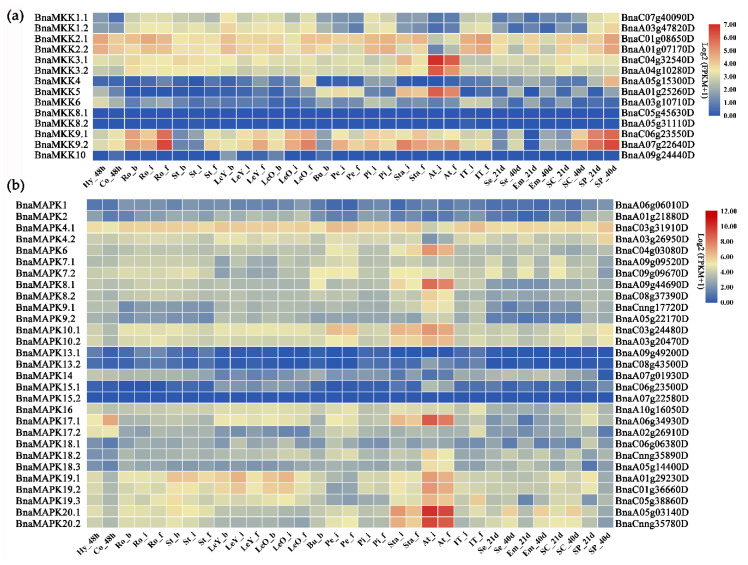
Spatial and temporal expression pattern of *BnaMKK* and *BnaMAPK* genes. (**a**) Heatmap of the expression profiles of *BnaMKK* genes in different tissues and developmental stages. (**b**) Heatmap of the expression profiles of *BnaMAPK* genes in different tissues and developmental stages. Hy_48h, hypocotyl 48 h after germination; Co_48h, cotyledon 48 h after germination; Ro_b, root at the bolting stage; Ro_i, root at the initial flowering stage; Ro_f, root at the flowering stage; St_b, stem at the bolting stage; St_i, stem at the initial flowering stage; St_f, stem at the flowering stage; LeY_b, young leaf at the bolting stage; LeY_i, young leaf at the initial flowering stage; LeY_f, young leaf at the flowering stage; LeO_b, mature leaf at the bolting stage; LeO_i, mature leaf at the initial flowering stage; LeO_f, mature leaf at the flowering stage; Bu_b, bud at the bolting stage; Pe_i, petal at the initial flowering stage; Pe_f, petal at the flowering stage; Pi_i, pistil at the initial flowering stage; Pi_f, pistil at the flowering stage; Sta_i, stamen at the initial flowering stage; Sta_f, stamen at the flowering stage; At_i, anther at the initial flowering stage; At_f, anther at the flowering stage; IT_i, tip of main inflorescence at the initial flowering stage; IT_f, tip of main inflorescence at the flowering stage; Se_21d, seed 21 days after flowering; Se_40d, seed 40 days after flowering; Em_21d, embryo 21 days after flowering; Em_40d, embryo 40 days after flowering; SC_21d, seed coat 21 days after flowering; SC_40d, seed coat 40 days after flowering; SP_21d, silique pericarp 21 days after flowering; SP_40d, silique pericarp 40 days after flowering.

Transcriptome deep sequencing (RNA-seq) dataset from the *B. napus* cultivar Zhongshuang11 in public database [38] was downloaded to characterize the expression profiles of *BnaMKK* and *BnaMAPK* genes in different tissues: Hypocotyls, cotyledons, roots, stems, young leaves, mature leaves, buds, petals, pistils, stamens, anthers, the top of inflorescences, seeds, embryos, the seed coat, and silique pericarp tissues. Expression (in Fragments Per Kilobase of transcript per Million mapped reads (FPKM)) was normalized as log_2_(FPKM + 1) prior to visualization as heatmaps, indicating that most *BnaMKK* and *BnaMAPK* genes were differentially expressed in distinct tissues and at various developmental stages (Figure 6). Indeed, *BnaMKK1*, *BnaMKK2*, *BnaMKK3*, and *BnaMKK9* were consistently expressed at high levels in most tissues, while *BnaMKK4*, *BnaMKK5*, *BnaMKK6*, *BnaMKK8*, and *BnaMKK10* showed little expression. Notably, *BnaMKK2.1*, *BnaMKK2.2*, *BnaMKK9.1*, and *BnaMKK9.2* were highly expressed in roots; *BnaMKK3.1*, *BnaMKK3.2*, *BnaMKK5*, and *BnaMKK9.2* in anther; *BnaMKK2.1*, *BnaMKK2.2*, *BnaMKK4*, *BnaMKK9.1*, and *BnaMKK9.2* in silique pericarp (Figure 6a). Likewise, *BnaMAPK1*, *BnaMAPK2*, *BnaMAPK13*, *BnaMAPK14*, and *BnaMAPK15* were expressed at low levels in most tissues. And our data also showed that *BnaMAPK4.1* was highly expressed in roots, stems, leaves, petals, pistils, stamens, and top of inflorescence. In addition, some genes were highly expressed in stamens and anthers, such as *BnaMAPK6*, *BnaMAPK8.1*, *BnaMAPK10.1*, *BnaMAPK10.2*, *BnaMAPK17.1*, *BnaMAPK19.1*, *BnaMAPK19.2*, *BnaMAPK19.3*, *BnaMAPK20.1*, and *BnaMAPK20.2* (Figure 6b). These data suggest that *BnaMKK* and *BnaMAPK* genes are largely ubiquitously expressed, but not followed the same expression patterns in distinct tissues and developmental stages, which would cause diverse pathways to involve in multiple processes during plant growth and development.

**Figure 7:**
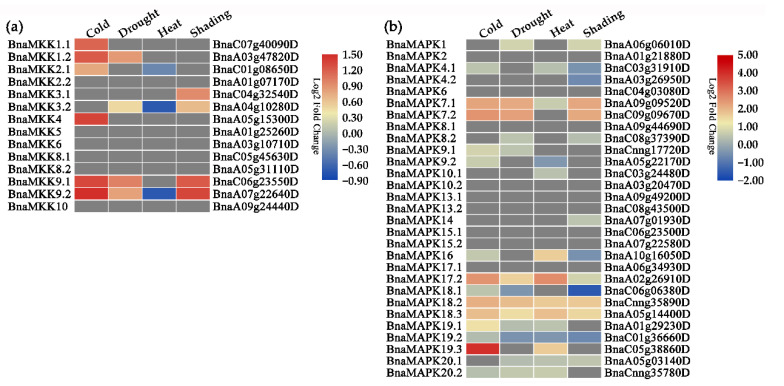
Heatmap representation of *BnaMKK* and *BnaMAPK* gene expression patterns upon exposure to cold, drought, heat, or shading stress. (**a**) Expression profiles of *BnaMKK* genes under four stresses treatments. (**b**) Expression profiles of *BnaMAPK* genes under four stresses treatments.

To assess the possible contribution of *BnaMKK* and *BnaMAPK* genes to abiotic stress responses, we next subjected 3–4-week-old plants from the *B. napus* cultivar Zhongyou821 to cold, drought, heat, and shading stresses and generated a new RNA-seq dataset. Several *BnaMKK* and *BnaMAPK* genes responded to cold, drought, heat, and/or shading stresses (Figure 7). For example, *BnaMKK1.1*/*1.2*/*4*/*9.1*/*9.2* expression was highly upregulated by cold stress, *BnaMKK9.1* expression by drought stress, and *BnaMKK9.1*/*9.2* expression by shading stress (Figure 7a). Likewise, *BnaMAPK7.1*/*7.2*/*17.2*/*18.1*/*18.2*/*19.1*/*19.3* were highly induced in response to cold stress, *BnaMAPK7.1*/*7.2*/*17.2*/*18.1*/*18.2* to drought stress, *BnaMAPK16*/*17.2*/*18.2*/*18.3* to heat stress, and *BnaMAPK7.1*/*7.2*/*18.2*/*18.3* to shading stress (Figure 7b). The same stresses also represent the expression of a number of genes: *BnaMKK3.2* and *BnaMKK9.2* were strongly downregulated by heat stress (Figure 7a), while *BnaMAPK18.1* was significantly downregulated by shading (Figure 7b). These data demonstrate that *BnaMKK* genes are sensitive to cold stress at the transcriptional level, while *BnaMAPK18.2*/*18.3* genes are sensitive to cold, drought, heat, and shading stresses. Based on their distinct transcriptional pattern, we hypothesize that *BnaMAPK7.1*/*7.2* play a role in cold, drought, and shading responses but not in heat stress. Similarly, *BnaMAPK17.2* transcript levels responded to cold, drought, and heat stresses but more weakly to shading stress, while the expression levels of *BnaMAPK17.1* showed no detectable or no significant change. Taken together, *BnaMKK* and *BnaMAPK* genes appear to exhibit preferential expression patterns in response to cold, drought, heat, and shading stresses.

### 2.5. Analysis of Interactions between BnaMKK and C Group BnaMAPK Proteins in Y2H Assays

To validate the *BnaMKK* genes identified by database search, gene-specific primers were independently designed based on the predicted *B. napus* genes to amplify the coding sequences of *BnaMKKs* from first-strand cDNAs and confirmed their sequences by DNA sequencing. All clones resolved into 14 *BnaMKK* independent cDNAs, with two copies each for *BnaMKK1* (*BnaMKK1.1* and *BnaMKK1.2*), *BnaMKK2*, *BnaMKK3*, *BnaMKK5*, and *BnaMKK8*, and one copy each for *BnaMKK4*, *BnaMKK6*, *BnaMKK9*, and *BnaMKK10*. The length of these sequences varied from 741 bp (*BnaMKK10*) to 1509 bp (*BnaMKK3.1*/*3.2*) (with a mean of 1074.4 bp), encoding proteins of predicted MW from 27.3 kDa (BnaMKK10) to 57.7 kDa (BnaMKK3.2) (with a mean of 40.0 kDa), and a pI ranging from 5.4 (BnaMKK3.1) to 9.0 (BnaMKK4) (with a mean of 6.4) (Table S3).

**Figure 8:**
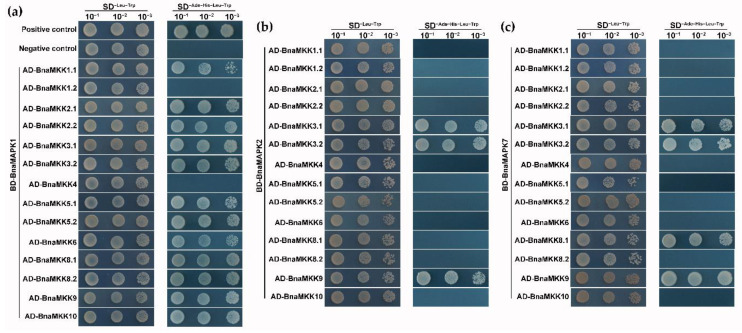
Yeast two-hybrid assays between BnaMKK and C group BnaMAPK proteins in the Y2H gold system. (**a**) Protein interaction analysis between BnaMKK family members and BnaMAPK1. (**b**) Protein interaction analysis between BnaMKK family members and BnaMAPK2. (**c**) Protein interaction analysis between BnaMKK family members and BnaMAPK7.

As much less is known about C group BnaMAPKs relative to the other groups, the coding sequences for *BnaMAPK1*, *BnaMAPK2*, and *BnaMAPK7* were cloned (the coding sequence for *BnaMAPK14* was attempted to clone, but failed). Then, BnaMKK proteins were fused to the GAL4 activation domain and BnaMAPK proteins to the GAL4-binding domain in Y2H vectors. After co-transformation into yeast (*Saccharomyces cerevisiae*) cells, transformants were selected on SD^−Leu−Trp^ medium and interactions were tested by growth on SD^−Ade−His−Leu−Trp^ medium. As shown in Figure 8, BnaMKK3.1, BnaMKK3.2 and BnaMKK9 interacted with BnaMAPK1, BnaMAPK2, and BnaMAPK7. By contrast, BnaMKK1.2 and BnaMKK4 showed no interaction with any of the C group BnaMAPK proteins tested here. BnaMKK8.1, but not its sister protein BnaMKK8.2, showed a strong interaction with BnaMAPK7 (Figure 8c). Notably, with the exception of BnaMKK1.2 and BnaMKK4, all other BnaMKKs proteins interacted with BnaMAPK1 (Figure 8a). Since ABA activates several MKK-MAPK signaling modules directly, the effect of adding 20 µM ABA to the yeast growth medium (SD^−Ade−His−Leu−Trp^ medium) were tested. However, most interactions were not affected by the presence of ABA, suggesting that external ABA is unlikely to directly activate BnaMAPKs via BnaMKKs (Figure S1). From these results, BnaMAPK1 appear to be more heavily connected to upstream MKKs than BnaMAPK2 or BnaMAPK7. In addition, because BnaMKK9 and BnaMKK3 interacted with the same set of partners here, these two MKK proteins may play a similar role in linking distinct signaling pathways converging from separate MAPK proteins.

## 3. Discussion

MAPK modules are major signaling pathways involved in biotic and abiotic stress responses in plants [39]. This signaling pathway bridges external stimuli and cellular responses and is evolutionarily conserved across eukaryotes [12]. In plants, upstream MAPKKKs phosphorylate and activate the downstream MKKs, which act as a middle converging point in MAPK cascades. MKKs then activate their own downstream MAPKs to transduce specific stimuli and evoke responses to stresses [5]. The *MKK* and *MAPK* gene families have been systematically investigated in *Arabidopsis* [7], rice (*Oryza sativa*) [40], maize (*Zea mays*) [41], wheat (*Triticum aestivum*) [42], diploid cotton (*Gossypium raimondii*) [43], barley (*Hordeum vulgare*) [44], tomato (*Solanum lycopersicum*) [45], cucumber (*Cucumis sativus*) [46], and purple false brome (*Brachypodium distachyon*) [47]. As the number and quality of plant genomes increase, an update on the *MKK* and *MAPK* gene families in *Brassica* species is warranted. In the current study, we carried out a comprehensive genome-wide analysis of the MKK and MAPK complement in five *Brassica* species. These results will provide a powerful theoretical foundation for future functional studies.

### 3.1. Characterization of MKK and MAPK Genes in Brassica Species and Their Evolution

In previous studies, BLASTP searches or queries with the keyword “MAPK” were employed to identify and characterize *MKK* and *MAPK* genes. Accordingly, 10 *MKK* and 20 *MAPK* genes were reported in *Arabidopsis* [7], eight *MKK* and 17 *MAPK* genes in rice [40]; six *MKK* and 16 *MAPK* genes in tomato [45]; nine *MKK* and 19 *MAPK* genes in maize [41]; six *MKK* and 14 *MAPK* genes in cucumber [46]; and 12 *MKK* and 16 *MAPK* genes in *Brachypodium distachyon* [47]. However, in most cases, initial reports of *MKK* and *MAPK* gene families in *Brassica* species were not reliable enough to allow a classification of these gene families. For example, 32 *BraMAPK* genes were initially identified in *B. rapa*, but the authors pointed out that 13 out of the 32 original *BraMAPK* genes in the Brasssica Database BRAD were inaccurate due to various errors [48]. Later, 15 *BraMKK* genes and 34 *BraMAPK* genes were reported in *B. rapa* that took the subgenomes into account [49]. Similarly in *B. napus*, Liang et al. identified seven *BnaMKK* genes and 12 *BnaMAPK* genes using *Arabidopsis* sequences as queries [50]. In the present study, *Arabidopsis* protein sequences of AtMKK and AtMAPK families were used to identify the full and up-to date complement of MKK and MAPK proteins in these five *Brassica* species. More accurate information on the quantity and structure of MKKs and MAPKs have been mined in our work, a total of 11 BjuMKKs and 25 BjuMAPKs from *B. juncea*, 14 BnaMKKs and 29 BnaMAPKs from *B. napus*, seven BniMKKs and 18 BniMAPKs from *B. nigra*, six BolMKKs and six BolMAPKs from *B. oleracea*, nine BraMKKs and 14 BraMAPKs from *B. rapa* were identified (Table 1). These MKKs and MAPKs were classified into four groups (A–D), as in *Arabidopsis* [7]. Notably, most MKK and MAPK family members appeared to be conserved in *Brassica* species, especially in *B. juncea* and *B. napus*, whereas multiple members were lost in *B. oleracea*. In the MKK family, MKK1/4/6 were conserved across all five *Brassica* species; MKK3/5/10 were conserved in *Brassica* species, excluding *B. oleracea*; and MKK8 was conserved in *Brassica* species but absent in *B. nigra*. In the MAPK family, no MAPK protein was absolutely conserved across all five *Brassica* species, but many were shared four out of the five species. For instance, MAPK1/2/9/10/13/15 were missing in *B. oleracea*; MAPK4/17 were absent in *B. nigra*; MAPK6 was not detected in *B. rapa*; MAPK19 was absent in *B. juncea* (Table 1). Unlike in previous report, all *B. napus MKK* genes existed as single copy and that *MKK7*, *MKK8*, and *MKK10* had no obvious *B. napus* orthologs [50]. In our study, 14 *BnaMKK* genes were identified, including the single copy genes *BnaMKK4/6/9/10* and the two copies genes *BnaMKK1/2/3/5/8*, showing that the number of most *BnaMKK* and *BnaMAPK* genes in *B. napus* has been retained or increased after whole-genome duplication (WGD) event. However, the sequence with high homology to *AtMKK7* was failed to identify in *B. napus* (Table S3), suggesting that *B. napus* has no clear ortholog for this gene or *BnaMKK7* gene may be lost after the WGD event. Furthermore, the phylogenetic trees and motif analysis indicated that BnaMKKs and BnaMAPKs were evolutionarily conservative, but functionally differentiated. Our data suggest that the retained and duplicated MKKs and MAPKs may have different functions during the growth and development process of the *Brassica* species considered here.

### 3.2. Functional Divergence of MKK and MAPK Genes during Growth and Development

Increasing evidence support a role for MKKs and MAPKs in plant growth and development [4,51]. For example, HvMKK3 regulated seed dormancy in barley [52], while the *Arabidopsis* ortholog AtMKK3 influenced blue light-mediated seedling development in *Arabidopsis* [53]. Our data showed that *BnaMKK3.1* and *BnaMKK3.2* were highly expressed in different tissues at different stages, including seed germination stages and anther development stages, suggesting that *BnaMKK3.1* and *BnaMKK3.2* may regulate seed and anther development. In *Arabidopsis*, *MKK9* was reported to control phosphate acquisition [54], nitrogen acquisition, anthocyanin accumulation [55], and leaf development [30]. In our study, *BnaMKK9.1* and *BnaMKK9.2* were expressed at high levels in young and mature leaves at the bolting, initial flowering and flowering stages, suggesting possible roles in leaf development. *MKK4* and *MKK5* were reported to modulate stomatal development [56], while *MKK6* appears to play an important role in meiotic cytokinesis during pollen development [31]. Here, the putative *B. napus* orthologs *BnaMKK4*, *BnaMKK5,* and *BnaMKK6* were seldom expression in most tissues and developmental stages, supporting the notion that these genes may not regulate growth or development or that any such regulation might occur at the post-transcriptional level. *AtMKK7* negatively regulates polar auxin transport [57], but the clear ortholog in *B. napus* have been unable to isolated to date. These data indicated that the loss of some *BnaMKKs* may lead to the functional redundancy of retained and increased *BnaMKKs*, enabling plants to maintain normal growth and development process. *BnaMAPK* genes exhibited distinct expression patterns across different tissues at various stages; indeed, 75.9% of *BnaMAPK* genes were constitutively expressed at high levels in all tissues and organs, with the remaining genes expressed at low levels in most tested tissues (Figure 6). Most research has thus far focused on MAPK3/6 and MAPK4 because of the ease by which their activation can be detected. In *Arabidopsis*, *MAPK3* and its homologous gene *MAPK6* were reported to control stomatal development [20,58,59], pollen development [60], anther development [61], and shoot branching [62]. *MAPK4* facilitates cell plate expansion and male-specific meiotic cytokinesis [33,63]. Similarly, *BnaMAPK4.1*, *BnaMAPK4.2* and *BnaMAPK6* were observed to highly express in all tissues at all stages. Moreover, *BnaMAPK10.1* and *BnaMAPK10.2* were also highly expressed in different tissues and at different stages, especially in petal, stamen and anther at the initial flowering and flowering stage. These data suggest that A group BnaMAPKs may play a more important regulatory role during growth and development. In addition, *MAPK12* was reported to be a novel negative regulator of auxin signaling [64], while *MAPK18* mediates cortical microtubule stabilization in *Arabidopsis* [65]. In the present study, three orthologs for *AtMAPK18* in *B. napus* were identified: *BnaMAPK18.2* and *BnaMAPK18.3* showed higher expression levels than *BnaMAPK18.1* in anther at the initial flowering and flowering stage, which indicated that there may be functional redundancy and functional differentiation among the members of *BnaMAPK18*. The clear ortholog for *AtMAPK12* was not identified, suggesting that MAPK12 function may have been lost and that of MAPK18 expanded in *B. napus*. The analysis of the tissue- or organ-specific expression patterns of *BnaMKK* and *BnaMAPK* genes indicated their functional redundancy and divergence during plant growth and development.

### 3.3. Functional Divergence of MKK and MAPK Genes in Response to Abiotic Stresses

Plants have evolved complex mechanisms to respond to adverse environmental conditions and defend themselves against abiotic stress, including cold, drought, osmolarity, heavy metals, and wounding, as well as biotic stress like pathogen and herbivore attacks [17]. Accumulating evidence has demonstrated that *MKK* and *MAPK* genes play key roles in the control of plant responses to biotic- and abiotic stresses and to phytohormones [15]. For example, *MKK1* mediates salt tolerance in rice [66], cotton [67], and *Arabidopsis*, and both *MKK1* and *MKK2* participate in plant responses to salt and cold stress [24] and to pathogen attacks in *Arabidopsis* [4]. Similarly, in our study, *BnaMKK1.1*, *BnaMKK1.2* and *BnaMKK2.1* showed high expression levels when plants were exposed to cold stress conditions. Since these three genes also showed high expression levels in growth and development process, we speculated that they play positive roles in the cross-talk between growth and development and abiotic stresses at the transcriptional level. For *BnaMKK2.2*, it expressed highly in the growth and development process, but not in response to stresses, which proved the functional differentiation of *BnaMKKs* in *B. napus*. *MKK3* was reported to mediate jasmonate [68] and pathogen signaling [69] in *Arabidopsis*, and responses to ABA and osmotic stress in maize [70]. Here, *BnaMKK3.1* expression responded to shading stress, and *BnaMKK3.2* to drought, heat, and shading stresses, although the amplitude of gene expression changes did not rise over 2-fold, suggestive of a weak transcriptional response for these two genes under these four stress conditions, possibly pointing to a major role in response to other types of stress. In cotton, *MKK4* regulates ABA, gibberellic acid, and hydrogen peroxide signaling [71]. *MKK5* itself is involved in responses to salt and drought stress [72] and both *MKK4* and *MKK5* play a vital role in response to wounding and herbivory attack by promoting ethylene production via the transcriptional upregulation of 1-Aminocyclopropane-1-Carboxylate Synthase in *Arabidopsis* [73]. By contrast, *BnaMKK4* only responded to cold stress, and *BnaMKK5* did not respond to any of the stresses tested here, which demonstrated that *MKKs* genes have different patterns in stress response in different plant species. Furthermore, *MKK9* plays a pivotal role in increasing alternative respiration in salt-treated *Arabidopsis* [74], and is also involved in rehydration-triggered ethylene production in roses (*Rosa hybrida*) [75]. Here, *BnaMKK9.1* and *BnaMKK9.2* were found to respond to cold, drought, and shading stress, and *BnaMKK9.2* was downregulated under heat stress, suggesting that *BnaMKK9.1* and *BnaMKK9.2* may play important roles in response to abiotic stress. Therefore, *BnaMKK3* and *BnaMKK9* may regulate the stress resistance more quickly than other *BnaMKKs* at the transcriptional level in *B. napus*.

Of the characterized MAPK family members, *MAPK3* transcript levels are induced by low temperature, osmotic stress, and mechanical stimuli in *Arabidopsis* [76], and show a rapid response to salt, heat, waterlogging, and wounding in *B. rapa* [48], as are the *B. rapa* genes *MAPK4* and *MAPK5* [48]. In *Arabidopsis*, *MAPK6* transcript levels are unaffected by low temperature, low humidity, touch, or wounding, whereas MAPK6 kinase activity is rapidly activated [77]. Here, we found no evidence for a *B. napus* ortholog of AtMAPK3 or AtMAPK5. In addition, *BnaMAPK4.1* and *BnaMAPK4.2* were expressed at low levels and *BnaMAPK6* expression remained constant across all stress treatments. About half of all *BnaMKK* genes (42.9%) and *BnaMAPK* genes (48.3%) were induced by cold stress. These numbers varied for *BnaMKK* expression in response to single stress treatment, but not for *BnaMAPK* genes, which exhibited close to 50% differentially expressed members: 28.6% *BnaMKK* and 44.8% *BnaMAPK* genes were induced by drought stress, 21.4% *BnaMKK* and 44.8% *BnaMAPK* genes were induced by heat stress, and 28.6% *BnaMKK* and 48.3% *BnaMAPK* genes were induced by shading stress. Furthermore, 7.1% *BnaMKK* and 17.2% *BnaMAPK* showed changes in transcript levels in response to all four stress conditions, and 21.4% *BnaMKK* and 41.4% *BnaMAPK* genes were responsive to any three stress conditions (Figure 7). For instance, *BnaMAPK7.1* and *BnaMAPK7.2* were expressed at high levels in response to cold, drought, and shading stress, *BnaMAPK17.2* was expressed at high levels after cold, drought, and heat treatment, while *BnaMAPK17.1* did not respond to any treatment, suggesting that the functions of BnaMAPKs may be redundantly specified in response to various stress conditions in *B. napus*. Taken together, these results indicate that *BnaMKK* and *BnaMAPK* transcript levels were generally modulated by growth and developmental signals and that the encoded proteins may play an important role in abiotic stresses like cold, drought, heat, and shading. These observations also highlight the possible extent of crosstalk between growth or developmental signals and abiotic stress responses.

### 3.4. Interaction Analysis between BnaMKKs and C Group BnaMAPKs

Plant MAPKs regulate numerous physiological responses by interacting with upstream and downstream protein components. A number of MKK-MAPK interaction partners have been characterized from many species, like *Arabidopsis* [78], rice [79], watermelon (*Citrullus lanatus*) [80], maize [41], and cotton [43]. The MKK4/5-MAPK3/6 pathway was reported to control stomatal development by phosphorylating the basic helix–loop–helix (bHLH) transcription factor SPEECHLESS [20,58,59]. The MKK7-MAPK3/6 cascade controls shoot branching by phosphorylating the auxin transporter PIN-FORMED 1 [62]. Similarly, the interaction between MKK6 and MAPK4 facilitates male-specific meiotic cytokinesis and root development in *Arabidopsis* [33,63]. Here, the interactions between BnaMKKs and the MAPKs BnaMAPK1, BnaMAPK2, and BnaMAPK7 were tested by yeast two-hybrid. BnaMAPK1 appeared to be more promiscuous than BnaMAPK2 or BnaMAPK7 based on the number of interactions detected: 12 for BnaMAPK1, three for BnaMAPK2 and four for BnaMAPK7, suggesting that C group BnaMAPK1 may play a more important role than BnaMAPK2 or BnaMAPK7. MKK3 in cotton and *Arabidopsis* were reported to stimulate the activity of C group MAPKs in response to ABA and pathogen attacks [43,69]. A previous report also determined that the MKK9-MAPK1/2 module mediates SA signaling in the *B. napus* cultivar DH12075 [50]. Here, our data showed that BnaMKK3.1, BnaMKK3.2, and BnaMKK9 interacted with the C group MAPKs BnaMAPK1/2/7.

A functional link suggested by interaction should be reflected in the expression pattern of the encoding genes; indeed, *BnaMKK3.1*, *BnaMKK3.2,* and *BnaMKK9* exhibited a similar temporal and spatial expression pattern to that of *BnaMAPK1*. By contrast, the expression of this subset of BnaMKK genes showed contrasting responses to stress, with *BnaMKK9* being highly expressed and *BnaMKK3.1* and *BnaMKK3.2* having a low expression level. We therefore speculate that the BnaMKK3.1/3.2/9-BnaMAPK1 module may be of significance for plant growth and development. BnaMKK9-BnaMAPK1 may also play a major role in response to abiotic stress, while BnaMKK3.1/3.2-BnaMAPK1 may regulate stress responses at the translational or post-translational levels, rather than at the transcriptional level. Moreover, *BnaMKK3.1* and *BnaMKK3.2* expression were especially highly expressed in anthers, suggesting that the BnaMKK3-BnaMAPK1 module may participate in anther development. Since *BnaMKK9* was also highly expressed in all tissues at all stages, especially in silique pericarps, we hypothesize that BnaMKK9 interacting with BnaMAPK2 may positively regulate seed development by controlling photosynthesis and nutrient biosynthesis in siliques. Cotton (*Gossypium hirsutum*) *MAPK7* was reported to regulate plant growth and development [81]. Interestingly, *BnaMAPK7* expression was higher than that of *BnaMAPK1* or *BnaMAPK2* in all conditions tested here, and followed a similar expression pattern as *BnaMKK3.1*, *BnaMKK3.2*, and *BnaMKK9*, suggesting that the BnaMKK3.1/3.2/9-BnaMAPK7 module functions in growth, development, and stress signaling. Therefore, it is very likely that C group BnaMAPKs have redundant functions during these signaling events, in agreement with prior research in *Arabidopsis* and cotton [69,81]. Besides the BnaMKK3.1/3.2/9-BnaMAPK1/2/7 module, the new interaction pairs BnaMKK8.1-BnaMAPK1/7 were also detected, although *BnaMKK8.1* was barely expressed across our two transcriptome datasets, suggesting that the function of the BnaMKK8.1-BnaMAPK1/7 module may not be regulated at the transcriptional level. Taken together, our results indicate the possible conservation of BnaMKK3/9-BnaMAPK1/2/7 cascades in response to growth, development, and abiotic stress. Our data also implied an inherent difference in signaling pathways between *B. napus* and other plant species, and highlighted the necessity to explore MKKs-MAPKs-mediated signaling pathways for use in genetic investigations and breeding of *B. napus*.

## 4. Materials and Methods

### 4.1. Genome-Wide Identification of MKK and MAPK Genes in Brassica

To comprehensively identify all *MKK* and *MAPK* members, whole-genome data for *Arabidopsis thaliana* (http://www.arabidopsis.org/) and five *Brassica* species (*B. juncea*, *B. napus*, *B. nigra*, *B. oleracea*, and *B. rapa*) (http://brassicadb.org/brad/) [82] were downloaded to construct a local protein database. Then, *Arabidopsis* MKK and MAPK proteins were used as query to search against *Brassica* proteins using BLASTP [83] with an e-value of 1 × 10^−5^ and minimum identity of 50% as the threshold. The HMMER3.0 program (http://hmmer.org/) [84] was employed to conduct a Hidden Markov Model (HMM) search using the serine/threonine-protein kinase-like domain (PF00069) as query with the e-value threshold of 1 × 10^−5^. The HMMER hits were then integrated with the BLASTP results and parsed by manual editing to remove redundant sequences. Those proteins displaying the consensus sequences as described by Chen et al. [47] were considered potential MKK and MAPK proteins. The theoretical pI and MW of the identified *Brassica* MKKs and MAPKs proteins were evaluated using Isoelectric Point Calculation of Proteins and Peptides (IPC) server (http://isoelectric.org/) [85].

### 4.2. Sequence Alignment, Phylogenetic Analysis, Chromosomal Location, and Gene Structure Construction

The full-length protein sequences for all MKK and MAPK proteins from the five *Brassica* species and *Arabidopsis* were aligned by the Clustal W program in the MEGA-X software [86] with default settings. The resulting file was used to generate a phylogenetic tree with the NJ method and 1000 bootstrap replicates in MEGA-X. The phylogenetic trees were visualized using FigTree v1.4.4 (http://tree.bio.ed.ac.uk/software/figtree/). The chromosomal positions of *BnaMKK* and *BnaMAPK* genes were provided by the Genoscope database and visualized with TBtools software [87]. For the gene structures analysis, the GTF annotation files for the *Brassica* genomes being considered here were downloaded and uploaded to TBtools to display gene exon–intron structures. Conserved motifs in BnaMKK and BnaMAPK proteins were analyzed using MEME suite v5.1.1 (http://meme-suite.org/tools/meme) [88] with the following optimized parameters: repetition number, any; minimum motif width, 6; maximum motif width, 50; maximum number of motifs, 10. MEME files were visualized with TBtools.

### 4.3. Plant Material and Growth Conditions

The black-seed doubled haploid (DH) inbred *B. napus* cultivar Zhongyou821 seeds were obtained from the Rapeseed Engineering Research Center of Southwest University in Chongqing, China (CERCR). The healthy seeds were sown in nutritious soil in the growth chamber (Model PGR15, Conviron, Controlled Environments Ltd., Winnipeg, MB, Canada) with 16 h light (25 °C)/8 h dark (20 °C) photoperiod, 800 μmol·m^−2^·s^−1^ light intensity and 75% humidity. Light was provided by a mix of cool white fluorescent tubes (Philips F72T/CW/VHO, Philips Lighting Compant, Somerset, NJ, USA) and Philips 60-W incandescent lamps (Philips Electronics Ltd., Markham, ON, Canada). The photosynthetically active photon flux density (PPFD) was measured with a quantum LI-185B radiometer/photometer (LI-COR, Inc., Lincoln, NV, USA). Plants were placed on the layer farthest from light banks in the growth chamber. For gene cloning, the leaves, roots and stems were harvested from 3–4-week-old seedlings at four-leaf stage, and frozen immediately in liquid nitrogen, then stored at −80 °C until RNA extraction. For RNA-seq analysis, four-leaf seedlings were used for cold, drought, heat, and shading treatments. For cold stress, plants were transferred to a growth chamber set to 4 °C and sampled 3 h after transfer. For drought stress, plants were sprayed with 10% PEG 6000 and sampled 3 h later. Heat stress was imposed by transferring plants to a growth chamber set to 42 °C; samples were collected 1.5 h later. For the shading treatment, plants were exposed to limited light conditions (300 μmol·m^−2^·s^−1^) and samples collected 12 h later. For the controls, seedlings were kept the same growth conditions as before. Samples were immediately frozen in liquid nitrogen and stored at −80 °C until sequencing.

### 4.4. Expression Analyses of the Two Families Genes in Rapeseed Using RNA-Seq Data

The RNA-seq dataset of *B. napus* cultivar Zhongshuang11 (BioProject ID: PRJNA358784) was described previously [38] and covers hypocotyl, cotyledon, root, stem, young leaf, mature leaf, bud, petal, pistil, stamen, anther, top of inflorescence, seed, embryo, seed coat, and silique pericarp at multiple developmental stages. Expression values (in Fragments Per Kilobase of transcript per Million mapped reads (FPKM)) of all *BnaMKK* and *BnaMAPK* genes were then normalized as log_2_(FPKM + 1) and submitted the results to TBtools to generate a heatmap. For RNA-seq analysis of stress responses, equal amounts of three individual plants leaves (by weight) were pooled as one biological replicate, and three independent biological replicates for each treatment and control were used in this study. Libraries were constructed using the NEBnext ultra RNA library prep kit (Illumina, San Diego, CA, USA) and sequenced at Beijing BioMarker Bioinformatics Technology Co., Ltd. (Beijing, China) on an Illumina platform. Raw RNA-seq data have been deposited at the Short Read Archive (SRA) repository (BioProject ID: PRJNA680826). The log_2_ (Fold-Change) values were calculated and submitted to TBtools to generate heatmaps. In all heatmaps, red and blue color represent increased or decreased expression levels, respectively, in comparison to controls; gray color indicates genes whose expression values were not significantly different from the controls (false discovery rate > 0.05).

### 4.5. Total RNA Isolation and cDNA Synthesis

Total RNAs from seedlings leaves, roots and stems tissues were respectively isolated using the RNAprep pure plant kit (Tiangen, Beijing, China) according to the manufacturer’s instructions. The RNA concentration and quality were determined on a Nanodrop 2000c spectrophotometer (Thermo Fisher Scientific, Waltham, MA, USA). One microgram of high-quality RNA was used for first-strand cDNA synthesis with the primescript RT reagent kit (Takara Bio, Mountain View, CA, USA), equal amounts (by volume) cDNA of each tissues, including leaves, roots and stems, were pooled as the mixture cDNA sample for gene cloning. *18S* primers were used to assess the quality of the cDNA preparations. Primer sequences are listed in Table S4.

### 4.6. Gene Cloning, Plasmid Construction, and Yeast Two-Hybrid Assays

The open reading frames (ORFs) for *BnaMKK* and C group *BnaMAPK* members were amplified with gene-specific primers (Table S4) from Zhongyou821 first-strand cDNAs and ligated into pGEM-T easy vector (Promega, Madison, WI, USA) following the manufacturer’s instructions. After sequencing and alignment against the reference genome, the confirmed *BnaMKK* ORFs were individually cloned into the pGADT7 prey vector, and C group *BnaMAPK* ORFs into the pGBKT7 bait vector.

Yeast two-hybrid (Y2H) assays were performed using the Matchmaker gold Y2H system (Clontech/Takara Bio, Mountain View, CA, USA), following the manufacturer’s protocol for co-transformation. The transformants were plated on SD^–Leu–Trp^ medium and grown for 2–4 days at 30 °C, then transferred onto solid SD^–Ade–His–Leu–Trp^ medium and grown for 3–5 days at 30 °C to observe protein interactions. pGBKT7-53 and pGADT7-T were used as positive controls, pGBKT7-Lam and pGADT7-T were used as negative controls. To determine the intensity of interaction between bait and prey, yeast cultures at stationary phase were diluted to 1:10, 1:100 and 1:1000 and spotted onto selective medium. The effect of ABA on BnaMKK-BnaMAPK interactions were also tested by adding 20 µM ABA to the selective SD^–Ade–His–Leu–Trp^ medium and spotting yeast cultures at stationary phase diluted 1:1000.

## 5. Conclusions

Our work identified 47 *MKK* and 92 *MAPK* genes from five *Brassica* species (*B. juncea*, *B. napus*, *B. nigra*, *B. oleracea*, and *B. rapa*), with 14 *BnaMKK* and 29 *BnaMAPK* coming from *B. napus*. Multiple protein alignments and phylogenetic analysis of *Arabidopsis* and *Brassica* MKKs and MAPKs helped classified them into 4 subgroups. Also, their chromosomal locations, exon–intron structure and conserved protein motifs were characterized. Our results suggest that genome duplication contributed to the expansion of the *BnaMKK* and *BnaMAPK* gene families. In the present study, we focused on the expression patterns of *BnaMKK* and *BnaMAPK* genes in different tissues and at developmental stages, as well as upon exposure to cold, drought, heat, or shading stress, revealing that *BnaMKK* and *BnaMAPK* genes may play key roles in growth and development and responses to environmental stress. Finally, 12 BnaMKKs-BnaMAPK1, 3 BnaMKKs-BnaMAPK2, and 4 BnaMKKs-BnaMAPK7 interactions were defined by yeast two-hybrid, of which the BnaMKK3.1/3.2/9-BnaMAPK1/2/7 module may play a conserved and key role during growth, development, and stress response. It will be interesting to explore their functions during abiotic and biotic stress responses.

## Figures and Tables

**Table 1:** Number of *Brassica* genes orthologous to *Arabidopsis* (*At*) *MKK* and *MAPK* genes identified from genome databases for five *Brassica* species.

	*AtMAPK*	Signature Motif	Group	*Bju*	*Bna*	*Bni*	*Bol*	*Bra*
MAPK kinases (MKK) family	*AtMKK1*	VGT(YP)YMSPER	A	1	2	1	1	1
	*AtMKK2*	VGT(YN)YMSPER	A	-	2	1	1	-
	*AtMKK3*	VGT(VT)YMSPER	B	2	2	1	-	1
	*AtMKK4*	VGT(IA)YMSPER	C	1	1	1	1	1
	*AtMKK5*	VGT(IA)YMSPER	C	2	1	1	-	2
	*AtMKK6*	VGT(YN)YMSPER	A	1	1	1	1	1
	*AtMKK7*	VGT(CA)YMSPER	D	2	-	-	-	-
	*AtMKK8*	VGT(FA)YMSPER	D	1	2	-	1	1
	*AtMKK9*	VGT(CA)YMSPER	D	-	2	-	1	1
	*AtMKK10*	VGT(CA)YMSPER	D	1	1	1	-	1
	Total			11	14	7	6	9
Mitogen-activated protein kinase (MAPK) family	*AtMAPK1*	T(E)Y	C	2	1	1	-	1
	*AtMAPK2*	T(E)Y	C	1	1	1	-	1
	*AtMAPK3*	T(E)Y	A	2	-	1	-	1
	*AtMAPK4*	T(E)Y	B	1	2	-	1	1
	*AtMAPK5*	T(E)Y	B	2	-	1	-	1
	*AtMAPK6*	T(E)Y	A	2	1	1	1	-
	*AtMAPK7*	T(E)Y	C	1	2	2	-	-
	*AtMAPK8*	T(D)Y	D	1	2	-	-	2
	*AtMAPK9*	T(D)Y	D	1	2	1	-	1
	*AtMAPK10*	T(E)Y	A	1	2	1	-	1
	*AtMAPK11*	T(E)Y	B	1	-	1	-	-
	*AtMAPK12*	T(E)Y	B	2	-	2	-	1
	*AtMAPK13*	T(E)Y	B	2	2	1	-	1
	*AtMAPK14*	T(E)Y	C	1	1	-	-	-
	*AtMAPK15*	T(D)Y	D	1	2	1	-	1
	*AtMAPK16*	T(D)Y	D	2	1	1	-	-
	*AtMAPK17*	T(D)Y	D	1	2	-	1	1
	*AtMAPK18*	T(D)Y	D	-	3	-	2	-
	*AtMAPK19*	T(D)Y	D	-	3	1	1	1
	*AtMAPK20*	T(D)Y	D	1	2	2	-	-
	Total			25	29	18	6	14

## Data Availability

Not applicable.

## Supplementary Materials

Supplementary materials can be found at https://www.mdpi.com/1422-0067/22/2/544/s1. Table S1: List of *MKK* and *MAPK* genes identified in *A. thaliana* and *Brassica* genomes; Table S2: Sequence length, molecular weight, and isoelectric point information for BnaMKK and BnaMAPK proteins predicted in *B. napus*; Table S3: Sequence length, molecular weight, and isoelectric point information for BnaMKK and C group BnaMAPK proteins cloned in *B. napus*; Table S4: Primers used to amplify and detect *BnaMKK* and C group *BnaMAPK* genes in this study. Figure S1, Yeast two-hybrid assays between BnaMKK and C group BnaMAPK proteins in the presence of 20 µM ABA. (a) Protein interaction analysis between BnaMKK family members and BnaMAPK1 with ABA. (b) Protein interaction analysis between BnaMKK family members and BnaMAPK2 with ABA. (c) Protein interaction analysis between BnaMKK family members and BnaMAPK7 with ABA.
