# Supplementary figures and images for: Genome-Wide Identification and Analysis of MKK and MAPK Gene Families in Brassica Species and Response to Stress in Brassica napus
Source: Int J Mol Sci. 2021 Jan 7;22(2):544. doi: 10.3390/ijms22020544 (PMC7827818; doi:10.3390/ijms22020544)

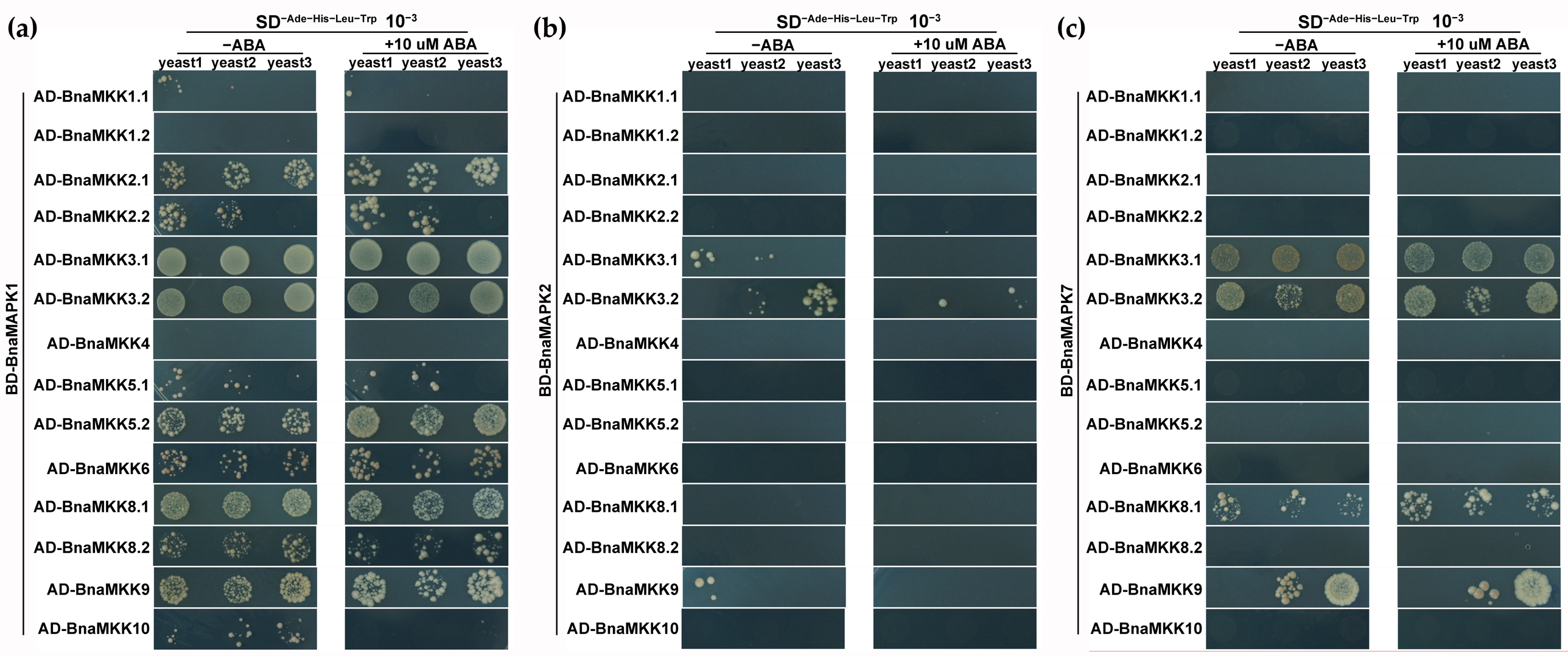

Supplement: Supplementary file 1 [file ijms-22-00544-s001.zip › FigureS1-proofed.tif]
